# Reverse-engineered exclusive enteral nutrition as induction therapy in pediatric Crohn’s disease: Effects on environmental toxin exposure

**DOI:** 10.1016/j.fct.2025.115773

**Published:** 2025-09-30

**Authors:** Elizabeth A. Reznikov, Melissa M. Melough, Dale Y. Lee, David L. Suskind, Shar Samy, James MacDonald, Theo K. Bammler, Sheela Sathyanarayana

**Affiliations:** a Department of Pediatrics, Division of Gastroenterology, Seattle Children’s Hospital and University of Washington, Seattle, WA, USA; b Department of Health Behavior and Nutrition Sciences, University of Delaware, Newark, DE, USA; c Department of Environmental and Occupational Health Sciences, University of Washington, Seattle, WA, USA; d Center for Child Health, Behavior and Development, Seattle Children’s Research Institute, Seattle, WA, USA; e Department of Pediatrics, University of Washington, Seattle, WA, USA

**Keywords:** Heavy metals, Inflammatory bowel disease, Nutrition

## Abstract

Exclusive Enteral Nutrition (EEN) can induce remission in Crohn’s disease (CD). We completed a pilot study using novel reverse-engineered EEN (RE-EEN), a whole food smoothie in place of commercial liquid formula (EEN) which contains food additives to improve shelf stability and palatability. In a four week trial with RE-EEN, we reported 80 % of patients went into clinical remission after four weeks. We hypothesized RE-EEN would decrease environmental toxin exposure through reduction of processing food intake. Biosamples were collected at baseline and at weeks two, four, and eight during the RE-EEN study. Urinary heavy metals were analyzed by inductively coupled plasma mass spectrometry, and urinary phthalate metabolites and melamine by liquid chromatography with tandem mass spectrometry. For our primary analysis, change in baseline was calculated using a paired *t*-test for week four. Analysis was also completed for all weeks on RE-EEN using a generalized least squares model. Results were expressed as fold change ± standard error mean. Paired t-testing demonstrated a statistically significant (p < 0.05) effect on molybdenum (Mo) with a fold change of 0.17 ± 0.15, an 83 % reduction following RE-EEN treatment. Our results suggested an effect of arsenic (As) with fold change of 0.23 ± 0.26 (p = 0.12), a 77 % reduction following RE-EEN treatment. Our results also suggested an effect of cobalt (Co) with a fold change of 3.12 ± 3.12 fold, a 212 % increase following RE-EEN therapy (p = 0.16). With inclusion of all weeks on RE-EEN, Mo and As were statistically significant (p < 0.05). Overall, we observed favorable shifts in urinary heavy metals by week four, and no effects were suggested in phthalate and melamine analysis. We saw increased precision in a sensitivity analysis when including all weeks for treatment. This is the first study to examine environmental toxicants in relation to whole foods smoothie diet in pediatric CD. RE-EEN dietary intervention shows promise in reducing chemical exposures and may contribute to CD remission. Notable limitations to this research include small sample size and absence of a control group. Further studies are necessary to assess the impact of RE-EEN diet on environmental toxicant exposure.

## 1. Introduction

Crohn’s Disease (CD), a form of inflammatory bowel disease (IBD), has seen a rapid rise in prevalence especially in the pediatric population, leading to significant morbidity (Bouhuys et al., 2023; Walton et al., 2024). The increase in CD in industrialized countries is likely linked to environmental factors in diets, such as agricultural contaminants, food processing contaminants, and increased consumption of processed foods high in sugars and fats that have been linked to intestinal inflammation (Rodrigues et al., 2024). Processed foods also increase exposure to environmental chemicals, including phthalates (Baker et al., 2024), heavy metals (Scutaraşu and Trincă, 2023), and melamine (Kim et al., 2023). Chemicals are introduced during the manufacturing, packaging, and food storage processes (Trasande et al., 2018).

Exclusive enteral nutrition (EEN) is the most widely accepted dietary therapy for CD (Reznikov and Suskind, 2023). EEN involves the use of a commercially made nutritionally complete liquid formula given exclusively in place of the usual diet. Commercial formulas used for EEN contain food additives as ingredients to improve shelf stability and palatability. However, exposure to these food additives and chemical contaminants that arise from food processing have also been shown to exacerbate inflammation within the intestines (Trasande et al., 2018). A novel dietary therapy using reverse engineered EEN (RE-EEN) was developed at Seattle Children’s Hospital (Lee et al., 2024). In RE-EEN, a home-blended smoothie was designed with whole foods to replace commercial formula. In our recent publication, 80 % of participants who were on RE-EEN achieved clinical remission (Lee et al., 2024). We built on the previously completed RE-EEN study and hypothesized that pediatric CD patients on RE-EEN had decreased environmental toxicant exposure compared to baseline due to RE-EEN formula being less processed and made with whole ingredients.

## 2. Methods

A pilot study was completed at Seattle Children’s Hospital with RE-EEN using whole food components to produce a nutritionally complete smoothie (Lee et al., 2024). Patients were given a whole-food formula recipe, as well as provided the food ingredients and a Vitamix™ blender to make the whole-food smoothie at home (Lee et al., 2024). Children with mild to moderate CD were asked to consume 100 % of total daily calorie needs from RE-EEN for four weeks, with the option to extend to eight weeks. Healthy pediatric controls were not given RE-EEN in this study. Ten subjects enrolled, eight completed the four week trial, and three continued through week eight. Randomization did not occur in the pilot study, as all individuals were on RE-EEN diet, and our outcomes were reported as change from baseline (week 0) for each subject. The protocol was approved by the Institutional Review Board at Seattle Children’s Hospital (IRB# STUDY00000619). The study was registered with ClinicalTrials.gov (NCT03508193). All patients/participants provided written informed consent or assent.

The urine samples analyzed for this paper were collected during the pilot study at baseline and weeks two, four and eight. Urine was collected into polypropylene specimen containers. All specimens were processed and aliquoted into high density polyethylene (HDPE) cryovials and stored at −80 °C. The concentrations of antimony (Sb), arsenic (As), aluminum (Al), cadmium (Cd), cobalt (Co), molybdenum (Mo), manganese (Mn), thallium (Tl), and tungsten (W) were measured in participants’ urine by inductively coupled plasma - mass spectrometry (ICP-MS) (Caldwell, 2015). Urinary concentrations of melamine were measured using liquid chromatography with tandem mass spectrometry (LC-MS/MS) (Zhu and Kannan, 2019). Urinary phthalate metabolites 4-MUB, MCPP, MEP, MEHHP, MiBP, MECPP, MBP, MEOHP, MHNP, MCOP, MBzP, MONP, MEHP, and MNP were measured in participants’ urine using LC-MS/MS. DEHP molar concentration was calculated using [(MEHP*(1/278.34)) + (MEHHP*(1/294.34)) + (MEOHP*(1/292.33)) + (MECPP*(1/308.33))] (Botelho, 2017).

All data points for urinary heavy metals, phthalates, and melamine were sorted by baseline and following RE-EEN dietary treatment. We examined all descriptive characteristics for the chemical concentrations. If more than 50 % of samples were at the limit of detection (LOD) or below, then results were excluded from further analysis. For results below LOD used in the analysis, we substituted *LOD*∕√2. Histograms were used to assess data distribution. Due to observed right skew, we *log*_2_ transformed data prior to statistical analysis. Tables 1 and 2 demonstrate data transformed back to linear scale after statistical analysis.

## 3. Results

Mo and As were above the LOD in 100 % of samples; Tl, Cd, Co in 70–80 % of samples; and W, Al, Mn, Sb in less than 50 % of samples. For phthalate concentrations, 4-MUB, MEP, MEHHP, MiBP, MECPP, MBP, MHNP, MCOP, MBzP, MNP, MEOHP and MBzP were above the LOD in 80–100 % of all samples; MEHP in 50 % of samples; and MCOP, MOiNP, MCPP, MiNP, MHiNP in less than 50 % of samples. Melamine was above the LOD in 80 % of samples. For phthalates, mean (±SD) concentrations (nanogram per milliliter (ng/ml)) ranged from 3.70 (±2.80) to 74.0 (±98.0) at baseline. Baseline mean (±SD) concentrations (ng/ml) for As, Cd, Co, Mo, and Tl were 19.0 (±31.0), 0.24 (±0.15), 0.50 (±0.30), 92.0 (±57.0), and 0.20 (±0.12), respectively. For melamine, mean (±SD) concentrations (ng/ml) were 13.0 (±15.0) at baseline.

Primary analysis provided in Table 1 used paired t-tests to assess fold change in urinary concentrations at week four compared to baseline. We saw a significant effect with Mo, and suggested effects with As and Co. Mo was reduced by 0.17 ± 0.15 fold (p < 0.05), an 83 % reduction following RE-EEN therapy. As was also reduced by 0.23 ± 0.26 fold (p = 0.12), a 77 % reduction following RE-EEN therapy. Co increased by 3.12 ± 3.12 fold, a 212 % increase following RE-EEN therapy (p = 0.16). The p-value can inform whether an effect exists, however we also report suggested effects that do not meet statistical significance criteria (p < 0.05) due to the observed percentage change that has clinical relevance in our model. We interpret these results in the context of a small, hypothesis generating clinical model.

The primary analysis was restricted to those subjects with a baseline and week four observation (Table 1). As a sensitivity analysis we compared treated versus baseline where treated included any post RE-EEN observation (weeks two, four, and eight) and included multiple observations for some subjects (Table 2). Unlike the paired *t*-test, which is restricted to just those subjects with both baseline and week four observations, considering any post-baseline observation as treated increases the number of observations, which may increase power. However, this comes at a cost of complexity, as any observations from the same subject may be correlated, which is problematic for a conventional linear model. In addition, the experimental batch, as well as age and sex of the subjects are no longer orthogonal to treatment (which they were for the paired *t*-test) and should be controlled for. We fit a generalized least squares (GLS) model, assuming compound symmetry for the covariance structure using the GLS function from the R nlme package. Mo and As were statistically significant (p < 0.05) with a fold change of 0.12 ± 0.07 and 0.30 ± 0.23, respectively. Co was insignificant in the GLS model (p = 0.20). No significant effects were seen or suggested in our phthalate or melamine analysis.

## 4. Discussion

Understanding how environmental chemicals in food impact IBD incidence remains a critical area of study. RE-EEN was developed as a whole foods alternative to current commercial formula used for EEN with a goal to reduce exposure to processed foods. By decreasing preservatives used for shelf stability, and bypassing commercial manufacturing and packaging processes, RE-EEN aims to minimize chemical contaminants. As such, we found overall reduced levels of Mo and As and higher Co in relation to RE-EEN treatment, and no significant or suggested changes were observed for phthalates and melamine.

The literature reports on several environmental contaminants that have an impact on intestinal inflammation and worsening of IBD severity as demonstrated in animal models (Chen et al., 2023). Heavy metals, phthalates and melamine were chosen for the RE-EEN model due to their potentially ubiquitous presence in processed foods occurring during food processing (which would be expected to be elevated in EEN). Of the chosen contaminants, specifically heavy metals have been most extensively studied in animal models and include Mn, Hg, Cd, Pb, and As (Chen et al., 2023) Clinical trials in adults with IBD have also demonstrated shifts in heavy metals levels associated with gastrointestinal inflammation. One epidemiologic study found that patients with relapsing CD had higher levels of Mn, As, Cd, Pb, and uranium (U), and lower levels of copper (Cu) (Stojsavljević et al., 2022). In adults receiving routine colonoscopy, patients with subclinical IBD on colonoscopy were found to have higher levels of sodium, potassium, and boron, and lower levels of zinc (Zn), U, Cu, and germanium (Ge) in hair samples (Rodríguez-Lago et al., 2024). A positive association was found with phthalate metabolites and CD activity in a cross-sectional study (Xiong et al., 2023) This clinical study was the first and only to demonstrate an association with phthalate metabolites and CD activity, and this was thought to be related to oxidative stress. No study to date, animal or human, has assessed the impact of melamine on CD.

Mo is an essential trace mineral naturally found in foods. Although it has been minimally studied in chronic disease models, *excess Mo* leads to respiratory and renal effects in animal models (ATSDR Molybdenum, 2020). Studies in animals also show that Cu levels affect Mo toxicity via Mo binding Cu-containing compounds (ATSDR Molybdenum, 2020). As discussed above, Cu levels are lower in active IBD (Stojsavljević et al., 2022; Rodríguez-Lago et al., 2024; Amerikanou et al., 2022). This suggests that the subjects in our study may have had improved Cu stores during RE-EEN treatment, as evidenced by their remission, which in turn could enhance Mo clearance. Another possible explanation for reduced Mo could be related to reduced dietary Mo intake. However, this is less likely given RE-EEN diet contained adequate sources of Mo (Lee et al., 2024).

In contrast to Mo, As has been studied extensively in many chronic disease models. As is both an essential and toxic heavy metal that can be found in contaminated soil, water, and food sources (ATSDR Arsenic, 2007). Ingesting As is known to cause circulatory and peripheral nervous system disorders, gastrointestinal disturbances (ranging from abdominal pain, nausea, vomiting, diarrhea, hematologic disorders), and skin changes (ATSDR Arsenic, 2007). Additionally, As is a recognized human carcinogen, linked to increase cancer risk in the liver, bladder, lungs, and skin (ATSDR Arsenic, 2007). Animal studies have demonstrated As is metabolized to toxic arsenic trioxide at the intestine leading to inflammation Chiocchetti et al. (2019); Zhong et al. (2021). In our study population, we observed decreased urinary As with RE-EEN treatment. This aligns with previously reported data on As levels in patients with relapsing CD (Stojsavljević et al., 2022). Furthermore, the RE-EEN smoothie diet did not contain seafood, poultry or grain-based food products commonly associated with dietary As exposure.

In contrast to Mo and As, our results suggest an increase in urinary Co following RE-EEN. Co is an essential element of cobalamin (vitamin B12) for which we are exposed to in the air, water and food sources, with food being the largest source of exposure (ATSDR Cobalt, 2024). At much larger concentrations of Co exposure, Co can have a hepatotoxic, thyrotoxic, neurotoxic, hemotoxic, and altered glucose homeostasis effect (ATSDR Cobalt, 2024). At a lower concentration, Co can lead to gastrointestinal upset. Respiratory effects can occur following inhalation exposure to Co (ATSDR Cobalt, 2024). Dietary Co is primarily found in animal-based foods, and the dairy in RE-EEN smoothie diet may explain an increased fold change in urinary Co. Contrary to the statistical significance seen with Mo and As, the GLS model did not strengthen the suggested effect for Co.

Overall, we observed favorable shifts in urinary heavy metals by week four RE-EEN treatment. We saw increased precision in a sensitivity analysis when including all weeks for treatment in our analysis. However, notable limitations to this research include small sample size and absence of a control group. To better understand the impact of environmental chemicals in RE-EEN, further studies are necessary, ideally through a well powered randomized controlled trial, and with longitudinal follow-up. In future studies, we would choose commercial formula as a control against RE-EEN. In addition, our study did not measure Cu levels. Future studies on RE-EEN would benefit from considering inclusion of Cu levels due to known interactions of Mo with Cu, and the findings demonstrated in this study.

In conclusion, this is the first study to investigate the relationship between environmental toxicants and a whole foods smoothie diet for achieving remission in pediatric CD. This pilot study indicates RE-EEN dietary intervention may hold promise in reducing chemical exposures. Our findings highlight the need for future research to explore if these exposures are in the causal pathway for reduction of inflammation or remission.

## Supplementary Material

1

Appendix A. Supplementary data

Supplementary data to this article can be found online at https://doi.org/10.1016/j.fct.2025.115773.

## Figures and Tables

**Table 1:** Fold changes from paired t-tests comparing baseline and week four RE-EEN therapy.

	Fold Change ± SEM	P-value
**Heavy Metals**		
As	0.23 ± 0.26	0.12
Cd	1.06 ± 0.81	0.92
Co	3.12 ± 3.12	0.16
Mo	0.17 ± 0.15	0.033**
Tl	1.34 ± 0.87	0.54
**Phthalates**		
MEP	1.16 ± 0.68	0.73
MEHHP	1.00 ± 0.78	1.00
MiBP	0.88 ± 0.47	0.74
MECPP	1.09 ± 0.53	0.79
MBP	1.05 ± 0.70	0.93
MEOHP	0.93 ± 0.70	0.89
MBzP	1.16 ± 0.82	0.77
MEHP	0.70 ± 0.29	0.29
DEHP Metabolites:	0.97 ± 0.50	0.92
**Melamine**		
Melamine	0.62 ± 0.28	0.19

(**) Values marked with are significantly different from baseline.

**Table 2:** Fold changes from the generalized least-squares (GLS) model comparing baseline and weeks two, four, and eight RE-EEN therapy.

	Fold Change ± SEM	P-value
**Heavy Metals**		
As	0.30 ± 0.23	0.04**
Cd	0.73 ± 0.41	0.43
Co	2.03 ± 1.54	0.20
Mo	0.12 ± 0.07	<0.0001**
Tl	1.13 ± 0.45	0.67
**Phthalates**		
MEP	0.99 ± 0.54	0.97
MEHHP	0.74 ± 0.39	0.42
MiBP	0.81 ± 0.35	0.50
MECPP	1.09 ± 0.37	0.70
MBP	0.75 ± 0.36	0.41
MEOHP	0.85 ± 0.41	0.63
MBzP	0.90 ± 0.42	0.75
MEHP	0.69 ± 0.20	0.087
DEHP Metabolites	0.84 ± 0.27	0.43
**Melamine**		
Melamine	0.67 ± 0.44	0.40

(**) Values marked with are significantly different from baseline.

## Data Availability

Data will be made available on request.
