# Supplementary figures and images for: Reverse-engineered exclusive enteral nutrition as induction therapy in pediatric Crohn’s disease: Effects on environmental toxin exposure
Source: Food Chem Toxicol. Author manuscript; Available in PMC 2025 Nov 24. (PMC12640686; doi:10.1016/j.fct.2025.115773)

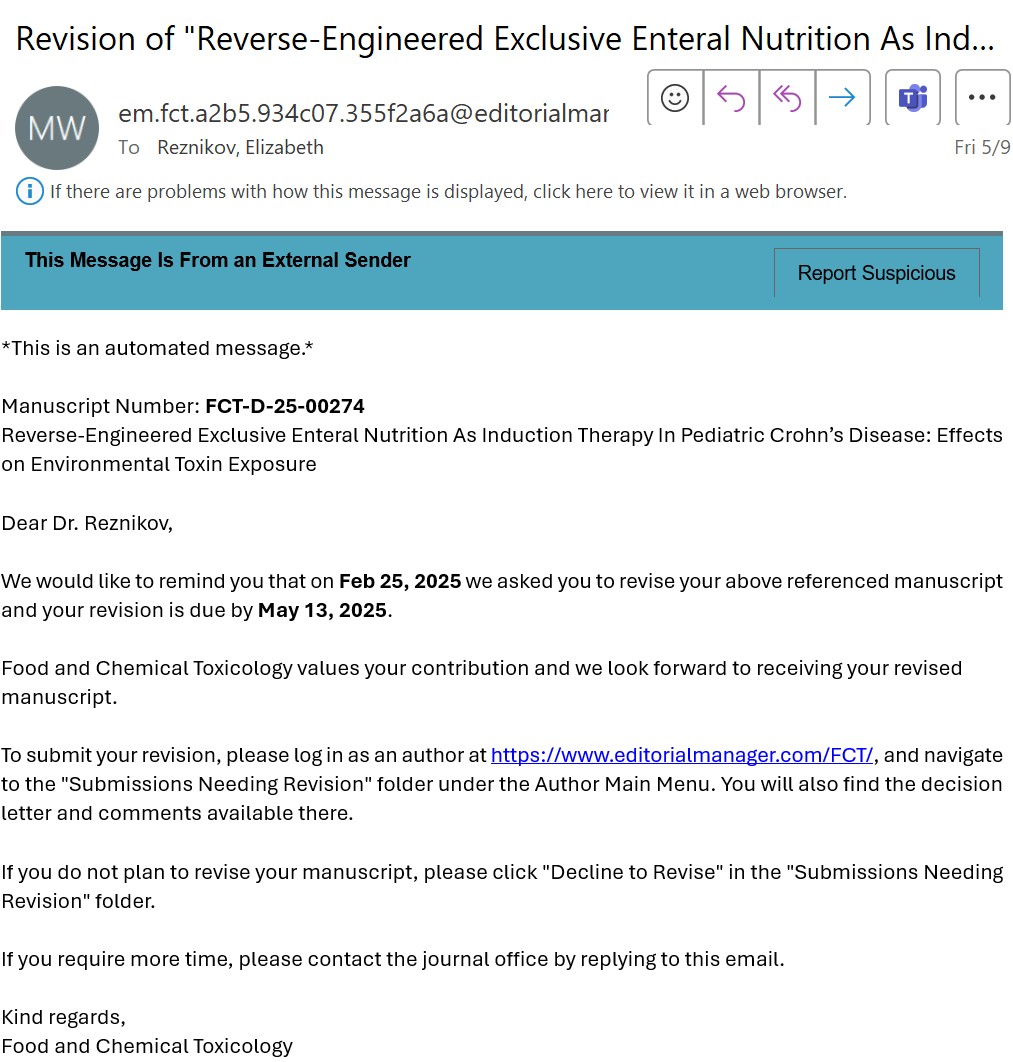

Supplement: 1 [file NIHMS2117717-supplement-1.jpg]
